# Acceptance of the Apple Watch Series 6 for Telemonitoring of Older Adults With Chronic Obstructive Pulmonary Disease: Qualitative Descriptive Study Part 1

**DOI:** 10.2196/41549

**Published:** 2023-12-26

**Authors:** Antonia Arnaert, Pia Sumbly, Daniel da Costa, Yuxin Liu, Zoumanan Debe, Sylvain Charbonneau

**Affiliations:** 1 Ingram School of Nursing McGill University Montreal, QC Canada; 2 Academic Affairs, Teaching and Research Directorate Montreal West Island Integrated University Health and Social Service Centre Montreal, QC Canada

**Keywords:** Apple Watch, chronic obstructive pulmonary disease, digital health, older adults, qualitative descriptive, technology acceptance, telemonitoring

## Abstract

**Background:**

The Apple Watch is not a medical device per se; it is a smart wearable device that is increasingly being used for health monitoring. Evidence exists that the Apple Watch Series 6 can reliably measure blood oxygen saturation (SpO_2_) in patients with chronic obstructive pulmonary disease under controlled circumstances.

**Objective:**

This study aimed to better understand older adults’ acceptance of the Watch as a part of telemonitoring, even with these advancements.

**Methods:**

This study conducted content analysis on data collected from 10 older adults with chronic obstructive pulmonary disease who consented to wear the Watch.

**Results:**

Using the Extended Unified Theory of Acceptance and Use of Technology model, results showed that participants experienced potential health benefits; however, the inability of the Watch to reliably measure SpO_2_ when in respiratory distress was concerning. Participants’ level of tech savviness varied, which caused some fear and frustration at the start, yet all felt supported by family and would have explored more features if they owned the Watch. All agreed that the Watch is mainly a medical tool and not a gadget.

**Conclusions:**

To conclude, although the Watch may enhance their physical health and well-being, results indicated that participants are more likely to accept the Watch if it ultimately proves to be useful when experiencing respiratory distress.

## Introduction

### Overview

Although the market for smartwatches has existed for many years, they have only recently been incorporated into the domain of health care delivery [1]. The advances in artificial intelligence and the use of sophisticated sensors capable of measuring vital signs such as heart rate (HR), respiratory rate, peripheral blood oxygen saturation (SpO_2_), and electrocardiograms are changing the self-monitoring landscape for patients while facilitating communication of their clinical data in real time with a health care provider. The Apple Watch Series 6 (Apple Inc) introduced an optical sensor that has been shown to reliably measure HR and SpO_2_ in patients with respiratory conditions such as chronic obstructive pulmonary disease (COPD) under controlled circumstances when compared with traditional commercial pulse oximeters [2].

In addition to the measurement accuracy, recent studies examining the feasibility of using an Android Wear smartwatch for continuous monitoring of patients with COPD have supported their potential for self-management, demonstrating the viability of the smartwatch-smartphone platform for early detection of acute exacerbations when worn consistently [3,4]. Although participants appreciated the inclusion of some biometric data, such as HR and activity level, they did not want a “passive monitoring device.” They wanted more feedback on their coughing and SpO_2_ values and expressed an interest in having access to COPD educational material, breathing exercises, and physical activity exercises to better manage their chronic condition [3]. Wearing the device may reduce feelings of anxiety associated with exacerbations and provide reassurance, yet in terms of usability, some older adults expressed concern about being a “prisoner of the numbers,” which they described as the “need to keep checking the device to know how they were doing, leading to the device dominating their lives” [4]. Other acceptability factors toward the smartwatch were related to the design of the device and its level of accuracy without significant technical issues. Specifically, patients with COPD desired a stylish device that does not label them as being sick and would enhance their sense of normalcy. In terms of affordability, these patients with COPD indicated that the cost of a smartwatch was a major concern, as many are living on a fixed income [4]. Similar concerns regarding the appeal of the smartwatch size, the interface design, and personalization were found to be important for older adults who tested an alternative smartwatch system for reporting pain, mood, fatigue, and sleep quality [5].

Specifically, regarding the Apple Watch, limited evidence exists on its acceptability and usability factors in older populations. To our knowledge, only Chen et al [6] explored the usability of the Apple Watch among 8 older patients in an emergency department with a recent fall using the Unified Theory of Acceptance and Use of Technology (UTAUT) conceptual model [7]. Results indicated that patients, after brief training, were able to wear and charge the Watch; however, their ability to engage with various features was influenced by their previous experience and comfort with technology, as well as the support and encouragement from family members and health care providers [6]. This significant gap in knowledge exploring the cognitive and behavioral aspects of acceptance of the Apple Watch as a device for facilitating health care delivery [5], is further compounded by a lack of understanding within the older adult population with health conditions such as COPD, where limitations and barriers to adoption associated with previous devices may be mitigated by the Apple Watch Series 6. In order to address this research gap, this qualitative descriptive study aimed to explore the acceptance and adoption of the Apple Watch among community-dwelling older adults with COPD for telemonitoring purposes using the Extended Unified Theory of Acceptance and Use of Technology (UTAUT2) model [8].

### The UTAUT2 Model

One of the most influential models to describe the factors influencing the acceptance of technology is the Technology Acceptance Model (TAM) [9], the subsequent UTAUT model [7], and its extended revision (UTAUT2) [8]. From the TAM, the beliefs of “perceived ease of use” (effort expectancy) and “perceived usefulness” (performance expectancy) are of primary relevance in explaining the attitudes that lead to “behavioral intention to use” (acceptance) and ultimately “actual use” [9]. The UTAUT added “social influence” and “facilitating conditions” as influencing “behavioral intention” and “use behavior” (actual use) [7], and the UTAUT2 added “hedonic motivation,” “price value,” and “habit” as intrinsic contributing factors, both including moderators such as age, sex, and experience that affect multiple pathways [8]. One of the driving factors in the revision of these models has been their limited applicability in fields such as health care, where additional determinants or moderators have been necessary to confer predictive value [10,11]. Therefore, the UTAUT2 model balanced extrinsic motivators such as “social influence” and “facilitating conditions” with intrinsic motivators to generate a more comprehensive predictive model, especially important when considering the adoption of personal devices such as smartwatches that are intended to be worn continuously.

## Methods

### Design, Sample, and Recruitment

This qualitative study was part of a larger mixed methods project that evaluated the use of an integrated telehealth nursing system to enhance patients’ health-related outcomes and reduce emergency room visits and hospitalizations, which took place from June 2020 to November 2021. Due to vendor-related issues, the project needed to switch to a different telemonitoring platform in June 2021, which presented the opportunity to offer an Apple Watch Series 6 to 10 participants. All 25 participants enrolled in the larger study were called and recruited for this qualitative study if they were interested in using the Watch for a period of 5 months. In order to be eligible for participation in the larger study, participants must (1) be diagnosed with COPD Global Initiative for Chronic Obstructive Lung Disease (GOLD) grade 2 or above, (2) have at least 1 emergency room visit or hospital admission in the previous year of enrollment, (3) be speaking English or French, (4) provide consent, (5) agree to be audio-taped, and (6) be followed at the COPD clinic of a regional acute hospital.

As shown in Table 1, participants had an average age of 71.6 (range 64-82) years. All participants were female except 2 (P1 and P4), 4 (P1, P5, P8, and P9) were legally married, 2 (P2 and P6) were divorced, and 1 (P7) was single. Of the 10 participants, all were retired except P6, who was unable to work. Participants’ levels of education varied: 1 (P6) had a middle school diploma, 7 (P2-P4 and P7-P10) had a secondary diploma of professional studies, and 2 (P1 and P5) had a bachelor’s degree. On average, participants were diagnosed with COPD for 6.4 (range 3-10) years, and 9 participants had an average of 2.2 hospitalizations since being diagnosed, except for P3, who had more than 20 hospitalizations since being diagnosed in 2012. Using the staging system of the GOLD, which considers a variety of factors, such as exacerbations, symptom severity, and forced expiratory volume, 3 people (P4, P6, and P10) were classified as GOLD grade 4 with “very severe COPD,” 3 (P1, P8, and P9) as GOLD grade 3 with “severe COPD,” and 3 (P3, P5, and P7) as GOLD grade 2 with “mild to moderate COPD” [12]. In terms of the ABCD assessment [13], all participants were labeled in the GOLD level D, meaning that they experienced more severe symptoms, such as greater dyspnea or exercise tolerance. All participants were provided with a Health Canada–approved digital fingertip pulse oximeter and an Apple Watch, and 7 (P1, P4, and P6-P10) received a project iPhone (Apple Inc).

**Table 1:** Sociodemographic data.

Participant	Age (years)	Sex	Marital status	Education	Native language	Year of diagnosis	GOLD^a^ level	Hospital admission, n
P1	82	Male	Married	Bachelor’s	French	2018	3D	2
P2	70	Female	Divorced	Secondary	English	2016	4D	1
P3	82	Female	Widowed	Secondary	French	2012	2D	>20
P4	68	Male	Widowed	Secondary	French	2016	4D	1
P5	68	Female	Married	Bachelor’s	English	2016	2D	1
P6	64	Female	Divorced	Middle school	French	2019	4D	4
P7	77	Female	Single	Secondary	French	2014	2D	1
P8	71	Female	Married	Secondary	English	2013	3D	4
P9	67	Female	Married	Secondary	English	2017	3D	3
P10	67	Female	Common law partner	Secondary	English	2016	4D	4

^a^GOLD: Global Initiative for Chronic Obstructive Lung Disease.

### Home Telemonitoring Nursing Intervention

Once patients agreed to use the Watch, the fourth author scheduled a 1-hour home visit to install and educate them on how to use the Watch and the commercial telemonitoring platform on their personal smartphone or a project iPhone. Participants were allowed to use all features on the Watch, download any apps, and were instructed to wear the Watch during awake hours. For a period of nearly 5 months, participants’ SpO_2_ and HR data were collected using the Watch, and they were asked to answer 5 specific COPD-related questions once every day using the platform. “How would you describe your level of shortness of breath today?” “How many times did you spit today?” “What color was your spit today?” “Was your spit thicker than usual?” “How often did you cough today?” Participants submitted their SpO_2_ and HR values using the fingertip pulse oximeters daily. Tailored patient education material was made available on the telemonitoring platform to complement their knowledge regarding lifestyle changes. When a measurement of clinical data was outside of expected patient-specific parameters, the telenurse would contact the patient, provide the necessary interventions, and, if needed, communicate with the interdisciplinary team at the COPD clinic of a local community hospital.

### Data Collection

After using the Watch for approximately 5 months, an individual semistructured interview was conducted with each participant during the months of September-October 2021. At the start of each interview, the purpose was re-explained, and a sociodemographic questionnaire was completed at the start of the larger study. The interviews were conducted in English or French through Zoom (Zoom Video Communications) by the first and fourth authors at a convenient time for the participant and lasted approximately 60-90 minutes. Both authors were present for all interviews. The interview with P3 was conducted in the presence of her daughter, as the participant had hearing problems. The interview guide included questions such as, “What did you know about the Apple Watch prior to the project?” “Please describe your initial thoughts when the Apple Watch was presented to you for telemonitoring purposes.” “How did you decide to participate in this Apple Watch project?” “What were the major difficulties you have encountered as of now regarding the Apple Watch?” “Can you explain how you have used the Watch over the span of one day?” “Which features have you used so far?” “How do you feel about the continuous monitoring of your SpO_2_ and HR?” To ensure alignment between the study aim and the interview questions, the guide was pilot-tested and validated with 2 key informants, and further refinements were made after the first few interviews [14].

### Data Analysis

All interviews were transcribed in the language of the interview and directly coded without translation, as all authors were fluently bilingual. Each transcript was manually analyzed using the inductive content analysis approach described by Elo and Kyngäs [15] and supplemented with field notes. A process of open coding was used to assign captions to segments of the transcripts. Codes were organized into categories and themes according to the UTAUT2 model, which captured similar concepts, from which descriptive statements were formed and supported with quotes. Several strategies were used to enhance the trustworthiness of the findings [16]. Peer debriefing and member checking were used to avoid possible biases and preconceptions and to ensure that the participants’ experiences were adequately represented. To address confirmability, dependability, and transferability, the interviewers wrote reflexive notes immediately after each interview, documented personal feelings and insights, and committed to a detailed description of the research methods, participants, and settings. Investigator triangulation through concurrent coding was also used throughout the analysis process to ensure credibility and confirmability.

### Ethical Considerations

Research ethics approval was obtained from the research ethics committee of an integrated health and social services center (Centre intégré universitaire de santé et de services sociaux de l'Ouest-de-L'Ile-de-Montréal) on August 16, 2019 (SMH #19-11). All participants signed the consent form before the start of the larger study, and written information was provided explaining the study purpose, participant involvement, the right to withdraw at any stage, and data confidentiality.

## Results

### Overview

Except for the component “price value,” all exogenous UTAUT2 constructs were used to explore the behavioral attitudes of older adults with COPD toward the adoption of the Apple Watch. Although the majority of participants had an overall positive judgment of the Watch, discrepancies in opinions are presented for each of the constructs. In terms of “performance expectancy,” some experienced health benefits and found the Watch more reliable, while others trusted the manual oximeter, especially in times of respiratory distress. The novelty of the Watch generated among many a mixture of emotions, from curiosity to fear and frustration, because of the practical issues with the Watch, which made it difficult to navigate the device and are covered by the construct “effort expectancy.” As discussed in the sections Social Influence and Facilitating Conditions below, participants’ families were supportive, and while some older adults were tech-savvy, others relied entirely on a family member to operate the device. Not owning the Watch was a hindrance to exploring new features. All participants perceived the Watch primarily as a medical tool, which is reflected in the construct “hedonic motivation.” Finally, as described in the section Moderators: Age and Sex below, male participants found it vital for older adults to learn and stay up to date with new technologies.

### Performance Expectancy

Specifically for the Watch, “performance expectancy” described the degree to which participants believed the device worked for the intended purpose of health monitoring and helped them to gain specific health benefits.

#### Gratitude for Health Benefits of the Watch

Although some participants (P5 and P9) felt fortunate to have the Watch and found it “absolutely genius,” their appreciation in terms of health benefits varied. Participants P3, P4, and P6-P8 used the “stand-up” feature and found that the Watch improved their overall health, as stated by P8: “I feel so much better physically. I haven’t thought of COPD since I got this Watch.” P4 continued: “My overall health and well-being has improved by 25%-30%. I won’t disobey the laws of the Watch; I will stand up if it tells me. That is a tool that someone like me needs.” In contrast, P2 had a different view: “I have a degenerative disease. The Watch gives me an idea of my SpO_2_ and HR, but it doesn’t improve my health.” Others (P5 and P6) were happy to better understand their sleep patterns. P10, however, ignored a message from the Watch suggesting that she had atrial fibrillation; “At the start, I was just using the Watch for the oxygen only [...] it kept telling me ‘You are having fibrillation problems, your heart is not beating properly,’ and I kept swiping [the message] aside thinking ‘oh it’s being sensitive for those kinds of things,’ but the reason for my hospitalization was due to my heart problems, and not my COPD.”

#### Reliability of Watch Versus Traditional Pulse Oximeter

Participants’ opinions were split regarding the use of the Watch or the traditional pulse oximeter for SpO_2_ measurements. In the occurrence of respiratory distress, the finger pulse oximeter was for some participants (P4, P7, P9, and P10) a more reliable option to capture their SpO_2_. P4, who had not yet mastered the use of the Watch, said: “The first thing I will get is the [traditional] oximeter because it is easier to put on my finger [than the Watch]. I do not understand the Watch well enough yet.” Due to the specific requirements of the positioning of the wristband, P9 was disappointed in the continuous monitoring of SpO_2_ and stated: “I had to manipulate the Watch 6 to 7 times, because it kept saying ‘unable to read, make sure your Watch is secure, comfortable,’ and I would move it and I would still get those messages, and I was annoyed by it.” On the contrary, others (P1, P2, P5, and P8) would vote in favor of the Watch. P1 stated: “I have the impression that the oximeter is less precise compared to the Watch because the manual oximeter we put it from finger to finger, hand to hand.” For P5 and P8, the Watch was more reliable because the manual one needed batteries. P8 said: “If you change the battery, you know that the light is brighter, and it is giving you a more accurate read. Whereas the technology of the Watch relies on your [wrist] positioning to get accurate measurements.” In terms of safety, P9 shared that the continuous monitoring of the Watch made her “feel even safer” but ended that she “felt safe with the [traditional] oximeter also.”

### Effort Expectancy

In terms of “effort expectancy” or “perceived ease of use” of technology, participants shared their experiences and challenges associated with the efforts needed to operate and navigate the Apple Watch.

#### Mix of Reactive Emotions Toward Watch Novelty

Except for 1 male tech-savvy person (P1) who expressed being “pretty up to date” with new technologies, the novelty of the smartwatch and its usage brought about a blend of reactive emotions for the majority of participants (P2, P5, and P6-P10), including curiosity, hesitancy, fear, frustration, anger, and stress. Although curious about the functionalities of new technologies, P9 felt anger when she did not know the workings: “I am curious [about new technologies] but there are things on my phone I don’t know how to do, and then I get angry.” However, being persistent, she described this process as fighting with the new piece of technology to “finally gain control over it.” In terms of being curious and not knowing what to do, P2 followed her daughter’s advice, saying, “I push a button till I get the right one. I am just using [the Watch], and I guess I become more confident.” Before being more confident, P2, P8, and P10 expressed initial hesitancy and fear, as P10 stated: “I am afraid I will do something that I won’t be able to correct [...]. I don’t like screwing up. I am sensitive about pushing buttons that I don’t know what is going to happen.” A total of 3 participants (P4-P6) did not find it difficult to use the Watch after receiving the instructions; they considered themselves quick learners. P5 said, “I am not a dummy. Once I learned how to [take the readings correctly], it was cool.” Although being able to learn fast, both female participants (P5 and 6) experienced stress and frustration when the Watch did not take the SpO_2_ measurement correctly at rest and when experiencing shortness of breath (SOB).

#### One’s Health and Manual Dexterity Affect Watch Usability

In terms of usability challenges, 5 participants (P2-P4, P6, and P10) stated that their personal health status and level of manual dexterity affected their ability to use the Watch. Although the Watch is a tool for monitoring health conditions, P6, who had severe COPD, mentioned that the Watch was unable to capture her SpO_2_ during frequent episodes of SOB. She shared: “Most of the time, when I am out of breath, the Watch cannot take my oxygen level. [...] I move a lot [when I am out of breath]. This tells me that the app doesn’t work.” Given that the Watch did not take her SpO_2_ when she needed it the most, she stated, “Is it worth it, I don’t know.” Participant P2, who did not feel well, indicated that her low energy level influenced her ability and interest in using and exploring various features of the Watch. The other 3 participants (P3, P4, and P10) expressed that there is a level of manual dexterity required to manipulate the Watch, as stated by P10: “I am pretty dexterous with my hands, but getting the Watch on and off, is a bit of a challenge with one hand.” This was validated by P3, who stated that the arthritis of her fingers affected her manual dexterity, which influenced the ease with which she could touch the Watch given its small size. Similarly, P4 stated that the size of his fingers affected his ability to use the device’s touch screen, saying, “It is so small compared to my fingers.”

#### Proper Wristband and Watch Placement Necessary

Participants shared usability challenges related to the Watch placement and the size and fit of the wristband. Improper placement of the Watch, which is a potential limitation of smartwatches, was a barrier for 3 participants (P5, P6, and P10) when attempting to measure their SpO_2_. In addition to the trouble of getting the Watch on and off, P10 found it challenging to find the correct placement of the Watch to ensure that the sensors were making skin contact. She stated, “It took months to know exactly where [the Watch] has to be on my arm. I have to do it a number of times [...] it is a little sensitive.” Participants P5, P6, and P9 experienced similar measurement reading issues until the telenurse suggested using a smaller wristband. P5 said: “With the other [larger size] wristband, it wasn’t reading. I was just trying to find the right position and thinking what I was doing wrong. But when switching to the smaller band, everything was Ok.” In terms of the wristband, P10 shared the following complaint: “The band has to be tight to take any readings, but the band is thick and rubber, and it makes you sweat. I would not wear this in bed.”

### Social Influence

“Social influence” was defined as the extent to which others in the participants’ social environment have impacted their decision to use the Apple Watch.

#### Positive Sentiments From Social Circle

Feedback from participants’ social circle to use the Watch varied; it fluctuated from a neutral response from loved ones to positive encouragement, curiosity, and 1 family member who decided to purchase a Watch for personal use. Except for the family of P9, who exhibited a neutral reaction, the social circle of the majority of participants (P3, P5, P6-P8, and P10) shared sentiments of being impressed and content that their loved one had access to the device. Friends of P5 stated: “They think it’s great. They want to know how they can get it, especially my friends think it’s phenomenal.” Along with the support of her daughter and friends, participant P3’s family physician also indicated that “it is a very a good idea” that the participant is part of this Apple Watch project. P6 said: “When I told them [3 daughters] about this new program, they said, ‘Take [the Watch], it’s good for you [...] they thought it was important that I was followed like this.’” For P8, the Watch health monitoring was “a family affair” and was seen as a source of comfort for her husband, stating, “If something happens to me, he could find out right away because I wear the Watch. It’s a comfort for him [...] he feels secure.” Furthermore, P8’s daughter was also happy to hear that her mother started using an Apple Watch. Specifically, “My daughter is happy that I get a Watch because it’s just a little security for the family [...] it is a positive thing for us, not just for me, for us.” For 1 participant (P2), her brother was influenced by her Apple Watch use and ended up purchasing one. She stated, “He bought it because he saw mine, he was interested when I told him.” Ultimately, despite being encouraged by family, P6 emphasized that “It’s not [my daughters] who decide, it’s me.” Similar reactions were voiced by the 2 male participants (P1 and P4).

#### Intergenerational Inspiration

Although participants tech savviness varied in using these devices, 3 participants (P6, P9, and P10) in particular spoke about the effect of intergenerational relationships in using novel technology. Participant P6 mentioned that her daughter influenced her curiosity about using different features on the Watch, while P10, who relied on her daughter to guide her with new technology, believed that she was “a little behind in technology.” When asked whether she felt a certain pressure to keep up with new technology, P10 stated, “No, only when my grandkids laughed at me,” and that the only reason she keeps up with technology is to “keep up with [the grandchildren] and to know what to buy for Christmas and birthdays.” Although P9 did not want to display any feelings of pride when using the Watch, she indicated, “I should [keep up with new technology] to stay in touch [with family and friends]. I love FaceTime and like to scroll Facebook.” Nonetheless, P9 stated, “I think a lot of older people are not comfortable enough with technology.” Furthermore, “the younger people want so much more information than my generation. I think if I had been able to have a Watch when I was 35-40, I would have jumped on it.”

### Facilitating Conditions

Various “facilitating conditions,” such as participants’ level of tech savviness, having the Watch on loan, and confidentiality issues, were influencing factors for acceptance of the Watch.

#### Personal Level of Tech Savviness

Participants’ comfort level and readiness with new technology either facilitated or impeded Apple Watch usage. Of the 10 participants, 2 (P8 and P10) identified themselves as not being tech-savvy and needing varying levels of support from family to manage their Watch; 5 (P2-P4, P6, and P9) were comfortable using the Watch after initial instructions; however, they may need help for technical problems; and 3 (P1, P5, and P7), who self-identified as being curious individuals and not fearful about the Watch, had the ability to use the device and find the necessary resources when encountering technical issues. Participants P8 and P10 completely relied on their respective daughters and husbands because of their fear of pressing buttons, deleting things, or breaking the Watch. P8 stated: “My husband sets me up and all I do is press that little button. I don’t have any patience with that [...] he usually sits beside me, and we do this together.” Although not very tech-savvy, participant P9 felt comfortable using the device by herself, saying, “I am comfortable with it now [after using it for 2 months]. I don’t use it for anything else [except for HR and SpO_2_]. I find it easy.” During the joint interview, the daughter of P3 shared that she teaches her mother how to use any new device, saying, “She learns superfast. I show her 1 or 2 times, and then she knows how to do it.” Participant P6, however, stated that her first instinct is to call her daughter when encountering a technical issue with the Watch. Although P4 described himself as “being resourceful,” which allowed him to use the Watch without any difficulties, he noted that his son lives at home and is available to him if needed. He said, “The Watch is more complicated than the telephone. I am techno, I am not an expert, but I can handle it [...]. At the start, my son showed me sometimes, but he is not patient.” The 3 tech-savvy participants (P1, P5, and P7) did not need support when they encountered any issues related to the Watch; they relied on themselves to research and resolve the issues on their own. Participant P7 stated: “I pulled out the little manual when I have a problem. I learn from my mistakes.” Participant P5 expressed her excitement about getting the Watch, and stated, “I always like to learn new things. This Watch was a new thing that I was going to learn as it was going to benefit me.” Her excitement and curiosity toward using the Watch were facilitated by her perception that she “can’t do damage to the Watch” because the “Watch can’t crash” like a computer can. Lastly, P1 is fearless about the technology: “I explored all applications [on the Watch]. I am absolutely not afraid, electronics and stuff like that, it’s part of our lives now.”

#### Apple Watch on Loan

Although participants were informed that they could use the Watch to its full capacity, 6 participants (P1, P3, and P5-P9) were reluctant and careful given that they did not own the device. Only P4 had no concerns about the Watch being loaned to him, stating, “No, I won’t eat it. Its waterproof [...] I have taken my bath sometimes and it never did anything. When I do the dishes, I don’t remove it either.” Contrarily, P6 felt that she needed to ask permission from the researchers before using the device in the shower. Participant P1 knew that the watch was waterproof “but did not dare” to test this feature. Furthermore, P8 stated, “I do not want to break your Watch,” and “When I have my own, I’ll play with it more than now. This belongs to you; I’m just doing the 2 things on it: the heart monitor and the oximeter.” Participants P6 and P7 expressed the same sentiment, stating that they use the Apple Watch just for the apps that are needed for the project and would not download more apps given that the Watch does not belong to them. During the project, participants P1, P2, P5, and P9 explored other features, such as the calendar, calculator, and photo apps, and were using the Watch to send and receive SMS text messages and emails and to answer phone calls. P9 shared: “The other day I answered the phone on it. I have never done that before, because I was in a store, and I just couldn’t get my phone, so I answered the call on the Watch.”

#### Watch Safety and Security

Only 3 participants (P1, P5, and P9) expressed their views on scams, security, and confidentiality issues. Participant P9 expressed her fear of being scammed through technology: “I’m afraid at some point I will push [a button] because I was scammed once with makeup on my iPad and it cost me like $800 because I pushed accept.” For P1, “hackers” accessing medical data was a concern. He said, “You have to have some fear of these things. There is still a level of confidentiality that is not yet 100%, I like my Watch but not banking things.” In contrast, P5 stated that she had no concern about sharing her COPD medical data with medical professionals, as it is “important for clinicians to see how much I am walking, how active I am [...] I know that the measures that are being monitored are all confidential anyways.”

### Hedonic Motivation

“Hedonic motivation” in the UTAUT2 refers to the fun and pleasure derived from using a new technology, for example, the Apple Watch.

#### The Watch, Fun but Primarily a Medical Tool

When asked if participants perceived the Apple Watch as a tool or gadget, all participants described this novel device primarily as being a medical tool. Participant P4 emphasized that “it is a tool, not a gadget. It has to be used intelligently. I used it 99% for the COPD; it is dedicated for that, and if I want to fool around, I go on Google and the TV.” Although both female participants (P5 and P8) stressed that the Watch is a physical health tool, they agreed that this smart device is fun. P5 stated, “I am using it for my benefit, but it is fun to look at. It is most definitely a physical health tool; it tells me every once in a while, stand-up, you have been sitting too long. [...] And the funniest thing is when I answered the telephone with my Watch.” She continued: “You should watch Dick Tracy; he could communicate with his police officers with his telephone watch.” In a similar fashion, P4 joked, “I feel like James Bond when I answer the phone using his Watch.” Participant P8 further shared: “It is our fun thing every day, we are enjoying the Watch. You lent me one of your toys and I am playing with it.” To conclude, P1 described the Watch as “a tool we cannot go without anymore.” He continued: “I have known the time that there was nothing like this. Now we are talking on the phone and seeing the person at the same time, it’s kind of science-fiction, but what do you want, we are there [today].”

#### The Watch, a Fashion Item

A total of 3 female participants (P3, P5, and P9) expressed how they perceived the Watch aesthetically. For participant P9, the Watch was seen as “big, perhaps because I have a small wrist, but I am not a good advertisement for the Watch, I would never buy one.” In contrast, participants P5 and P9 mentioned that it was not a physical change, as they are both accustomed to wearing watches. Participant P3 said, “I am a Watch lover. I see [the Apple Watch] as an ordinary watch. But if I go out to a big party like before, I think I would take it off and put on a nice watch.” Participant P5 furthered this sentiment when describing the Watch as being “cute and fashionable” and described herself as “trending,” given that “larger watches are trending right now.” In terms of the color of the Watch band, she joked, “I wish it was a little fancier black. Black is safe because white would get dirty.”

### Habit

Although participants only used the Watch for about 2 months, the new theoretical construct “habit” added to the UTAUT2 describes the extent to which they incorporated the device into their daily routines and their awareness of its capabilities.

#### Awareness of Watch and its Features

Except for P4, who did not have a computer or smartphone before participating in the project, all participants had various experiences with computers or mobile devices. Participant P1, who called himself a “neophyte in electronics,” said: “I use the phone for a million things. I talk to our daughters, I text, send, and receive emails. I go on Skype, twitter, and all of that.” Others, like participants P2 and P6, have used computers at work; as stated by P2: “I have been somewhat self-taught, I learned to use Excel. I have worked as an accounting technician and managed to work with the computer software there.” Overall, 3 of the 10 participants (P5, P8, and P10) had a FitBit, and P5 joked: “My children bought a FitBit and I said, ‘I don’t need it anymore, I have my Apple.’” Participant P8 added: “My husband bought me a FitBit, a pain in the butt. I don’t like it at all. It just beats 5000 more steps and 5 stars and whistles. I don’t like bells and whistles. It is simple [with the Watch], I push a button and get my HR, I push a button and get my oxygen.” Participants’ knowledge, awareness, and experience with the Watch greatly differed. Participant P4 mentioned: “I have heard talking about the smartwatches on television, but I had never one in my hands.” Others (P1, P3, P6, P7, and P9) have seen their children, family members, or friends using their Watch for health and entertainment purposes; however, only P9 mentioned knowing that the Watch could monitor one’s HR and SpO_2_.

#### The Watch, Part of the Daily Routine

Although there was some variability, all 10 participants incorporated the Watch into their daily routines. They were wearing the Watch throughout the day, unless it needed to be charged or they took a shower, and 3 of them (P2, P5, and P6) used the Watch at night. P4 admitted that incorporating the Watch into his daily routine was an effort and commitment, and for P6 and P9, who did not wear a Watch for a long time, it was often annoying at the beginning. Participant P2 shared: “It sure changes the routine a little bit, but it fit in what I was doing before.” Participant P10 described herself as a “creature of habit” and stated that the device became “part of her daily routine regime.” The rigidity of her daily routine was evident as, even when hospitalized, P10 continued using the Apple Watch to measure her SpO_2_ daily.

### Moderators: Age and Sex

Despite their differences as to the awareness of the Watch and its related features before their project enrollment, both male participants (P1 and P4) were comfortable using the device compared to many female participants. Although P4 did not own a computer or a smartphone, the Watch gave him an opportunity to learn, saying, “It gave me a chance to wake up and educate myself.” The idea that the Watch was an opportunity to learn was shared by P5, who believed that “anyone should learn about new technologies as long as they know the benefits.” This sentiment was supported by P1, who found it important for older adults to stay up to date with technology but simultaneously acknowledged that technology has its limitations. He concluded: “I can survive without technology, it is not a necessity, but it can facilitate things, such as communication.”

## Discussion

### Overview

Although COPD is a degenerative disease, findings showed that the use of various Apple Watch monitoring apps, such as “Sleep Cycle,” “Stand Up,” etc, may improve an individual’s physical health and overall well-being. People were encouraged by family to use the Watch; it was a fun experience, and some were eager to learn to use the wearable and felt that older adults should remain up to date with new technologies. Despite the benefits, the findings brought out concerns related to Apple Watch use in older adults with COPD. Hence, the following two points warrant discussion about the Watch: (1) feasibility of measuring SpO_2_ values of patients with COPD in their natural environment; and (2) perceived value of the Apple Watch.

### Feasibility of Measuring SpO_2_ Values in Patients With COPD in Their Natural Environment

As indicated above, the Apple Watch Series 6 is a reliable device for obtaining HR and SpO_2_ in patients with lung diseases under controlled conditions [2]. More specifically, the study observed strong positive correlations between the Apple Watch device and commercial finger pulse oximeters [2]. However, our study participants with severe COPD who used the Watch in real life shared their stress and frustration as the SpO_2_ app did not work when experiencing SOB. This finding was supported by Spaccarotella et al [17], as they were unable to measure SpO_2_ with the Watch for 8 of their participants with lung or cardiovascular diseases. The authors believed that skin perfusion and anatomical variability of the wrists could be a plausible reason for this defect [17]. Interestingly, the device’s makers, Apple Inc [18], supported these claims and stated, “Even under ideal conditions, your Apple Watch may not be able to get a reliable blood oxygen measurement every time. For a small percentage of users, various factors may make it impossible to get any blood oxygen measurement, including motion, watch placement on the wrist, skin temperature, skin perfusion, permanent or temporary changes to the skin such as tattoos, and HR above 150 bpm while at rest” [18]. Essentially, Apple Inc designed the oxygen monitor for general fitness and wellness purposes and is not considered a Class II medical device by the Federal Drug and Administration (FDA), which does not require clearance. This information-only offering approach of all health-focused apps on the Apple Watch is in contrast with Apple’s electrocardiogram feature, which was designed and marketed as a medical-grade feature and obtained FDA clearance in 2020.

Despite the instructions and suggestions provided by the company on how to use and measure SpO_2_ values and the fact that the blood oxygen monitor is a “wellness feature” and not intended for medical diagnostic use, the following questions remain: “Is it appropriate for patients with lung diseases, such as COPD, to use the Watch oxygen monitor in daily situations? Does it cause more harm than good for these patients?” Despite the fine-print warnings, people will primarily use the Watch SpO_2_ feature as a medical tool, as indicated by our study participants. The Watch was not a gadget but a tool to monitor their respiratory status and communicate with the telenurse. In cases of inconclusive results, some participants would repeatedly retake their measurements, which may cause them unnecessary anxiety. Dr Rosman, a cardiologist who is currently studying the effects of devices on anxiety, indicated that the use of the Watch “opens the door to a lot of questions and concerns from patients that are currently being unaddressed” [19]. In addition, Dr Friedman, a cardiologist, strongly emphasized that a distinction must be made between measurements for wellness and medical purposes [19].

While Apple Inc is marketing the Watch as a device with medical functions while insisting it is not a medical device, patients who are not well versed in the limits and particularities of the pulse oximeter feature can develop overreliance and, as a result, experience negative health effects. A common consequence may be that people will call their health care practitioner more often due to falsely low SpO_2_ readings; however, a more concerning and dangerous scenario would manifest when the Watch provides false reassurance and people do not seek health care advice [20,21]. Although more research will be needed on continuous SpO_2_ measurements using wrist-worn wearable devices for patients with COPD, 1 observational study, which tested the WristOx_2_ pulse oximeter among patients with clinical stability, concluded that significant SpO_2_ fluctuations occurred between and within multiple days and nights for their study patients [22]. They suggested that knowing these continuous SpO_2_ values is key to setting tailored SpO_2_ alerts for patients using telemonitoring, as no protocols are available [23]. Despite using a traditional or smart wearable pulse oximeter, physicians are often debating the need for a pulse oximeter at home for people with COPD. It may be useful if supplemental oxygen therapy is used, yet a home pulse oximeter is not a substitute for COPD management, nor does it replace the patient’s own personal assessment of their situation [24].

### Perceived Value of the Apple Watch

Despite the SpO_2_ measurement challenges and those related to the wristband and placement of the Watch, some study participants were inclined to use the Apple Watch. According to Hsiao [25], this technology adoption behavior may be attributed to the perceived value of the wearable [26]. First, the health benefits gained by using the Watch were critical for some study participants’ acceptance of the technology. Second, the remark made by participant P5, who did not want her FitBit anymore as she had “an Apple” now, demonstrated that the Apple brand name and its quality and performance may influence one’s adoption intention toward the Watch. Third, in terms of social value, some study participants were influenced and needed support from family to use the Watch, and, in turn, they influenced other loved ones to purchase a personal device. Chen et al [6] confirmed those findings, as their participants also needed support from family to navigate some of the advanced features of the Watch. Lastly, smartwatches are known to be fashion accessories, which may also enhance adoption intentions [27]. Participant P5, in particular, admitted that wearing a big Apple Watch is trendy, cute, and fashionable nowadays. When the Apple Watch, which is an extension of one’s iPhone, made its debut in 2015, it was seen as a fashion accessory [28]. However, as mentioned by our study participants P1 and P5, it makes things more convenient now, for example, answering phone calls. In addition to the health and fitness tracking functions of the Watch, the value of convenience may perhaps not be underestimated for older adults or people with disabilities [29], and, as such, increase its perceived value.

### Study Limitations and Recommendations

The fact that the Apple Watches were lent to participants can be seen as a limitation, as some did not explore their full capabilities out of fear of damaging the device. Second, participants only had 5 months to use their Watch, and in addition, the majority were female users. These factors may also have influenced the acceptance of the technology in terms of “perceived usefulness” and “ease of use.” Lastly, study participants could be considered to have a baseline level of curiosity given their interest in and acceptance of using the Watch in this project. The actions of our participants demonstrated their comfort or interest in technology, even though many did not self-identify as being “tech-savvy.” Although different strategies were used to enhance trustworthiness, the researchers are aware that subjectivity is always present in qualitative research; however, it may become an inspiration for further research. Knowing that a widespread uptake of wearables among older adults with various chronic conditions may be expected, more research is needed regarding the acceptability of the Watch and foremost on the “black box of wearable algorithms” for health monitoring [30].

### Conclusions

The findings of this study have shown that the UTAUT2 is a suitable model to obtain valuable insights into how older adults with COPD experienced the use of the Apple Watch for telemonitoring purposes. Despite their different views, participants were inclined to use the Watch for health monitoring and communication purposes, and some enjoyed interacting with the novel device. Nonetheless, results have shown that the Watch must be used with caution given that the SpO_2_ feature is not intended for medical use and does not function reliably when patients experience SOB. With the uptake of these smartwatches, it is imperative that health care providers are aware of these limitations and remind their patients of the current limits of the Apple Watch.

## Abbreviations

COPD: chronic obstructive pulmonary disease
FDA: Federal Drug and Administration
GOLD: Global Initiative for Chronic Obstructive Lung Disease
HR: heart rate
SOB: shortness of breath
SpO_2_: blood oxygen saturation
TAM: Technology Acceptance Model
UTAUT: Unified Theory of Acceptance and Use of Technology
UTAUT2: Extended Unified Theory of Acceptance and Use of Technology

## Data Availability

The data sets generated and analyzed during this study are not publicly available due to their confidential nature but are available from the corresponding author on reasonable request.
